# Effects of Mirtogenol^®^ on ocular blood flow and intraocular hypertension in asymptomatic subjects

**Published:** 2008-07-10

**Authors:** Robert D. Steigerwalt, Belcaro Gianni, Morazzoni Paolo, Ezio Bombardelli, Carolina Burki, Frank Schönlau

**Affiliations:** 1 Department of Biomedical Sciences, University of Chieti-Pescara, San Valentino, Italy; 2 Indena S.p.A. Scientific Department, Milan, Italy; 3 Horphag Research (UK) Ltd., Geneva, Switzerland; 4 Horphag Research (UK) Ltd., London, United Kingdom

## Abstract

**Purpose:**

The most important variable risk factor for developing glaucoma is intraocular hypertension. Timely lowering of high intraocular pressure (IOP) significantly lowers the likelihood of developing glaucoma. The aim of this study was to evaluate the effects of the food supplement Mirtogenol^®^ (Mirtoselect^®^ and Pycnogenol^®^) on IOP and ocular blood flow in a product evaluation study.

**Methods:**

Thirty-eight asymptomatic subjects with intraocular hypertension were either given Mirtogenol^®^ (20 subjects) or were not treated (18 subjects). The visual acuity, IOP, and ocular blood flow were measured at two, three, and six months.

**Results:**

After two months of supplementation with Mirtogenol^®^, the mean IOP decreased from a baseline of 25.2 mmHg to 22.2 mmHg. After three months of treatment with Mirtogenol^®^, the IOP was significantly lowered compared to that of untreated controls (p<0.05) to 22.0 mmHg. No further improvement was found after six months. Nineteen of the twenty patients taking Mirtogenol had a decreased IOP after three months. Only marginal effects on the IOP were found in the 18 control subjects. No side effects were observed. Ocular blood flow (central retinal, ophthalmic, and posterior ciliary arteries) improved both in the systolic and diastolic components as measured by Color Doppler imaging. After three months of treatment, the improvement of ocular blood flow was significant as compared to both baseline and control group (p<0.05).

**Conclusions:**

An improved ocular blood flow may contribute to the prevention of glaucoma. The results of this study indicate that Mirtogenol^®^ may represent a safe preventative intervention for lowering the risk for developing symptomatic glaucoma by controlling IOP and improving ocular blood flow.

## Introduction

The age-related decline of vision is caused by physiologic and pathologic degenerative processes. There are four major causes for age-related visual loss: cataract, age-related macular degeneration (AMD), retinopathy, and glaucoma [1]. Due to the possibility of surgery for cataracts, AMD is now the leading cause of blindness in developed countries. With the global diabetes epidemic, retinopathy is expected to represent a growing problem in the future. The second leading cause for blindness is glaucoma of which different forms exist with a progressive optic neuropathy as the common denominator. Primary open-angle glaucoma (POAG) is one of the most common forms of glaucoma [2].

Except for cataract, there is no real treatment available for AMD, retinopathy, and POAG. Slowing of the progression of these diseases remains the only resort. With the aging of the population, prevention of eye diseases receives considerable attention. Nutritional intervention, predominantly with antioxidants, has been suggested to lower the incidence of degenerative eye disorders. A study of 35,551 female health professionals over a period of 10 years has found a significant risk reduction for developing cataract with a higher dietary intake of lutein/zeaxanthin and vitamin E from food or dietary supplements [3]. Epidemiologic studies have clearly demonstrated that dietary vitamin C and E as well as zinc and the carotenoids, lutein and zeaxanthin, have a protective effect in AMD and may slow disease progression [4]. Protection from developing AMD is also suggested from dietary omega-3 fatty acids [5]. For diabetic retinopathy, the best risk reduction is a tight blood glucose control [6]. Specific flavonoids such as the standardized bilberry extract, Mirtoselect, and the French maritime pine bark extract, Pycnogenol, have been successfully tested for counteracting progression of retinopathy [7-9].

An effective dietary prevention for reduced progression of POAG has so far not been identified. The relation between dietary antioxidant intake and risk of POAG was examined in participants over 40 years of age in the Nurse’s Health Study (n=76,200) and the Health Professionals Follow-up Study (n=40,284) [10]. No significant association between antioxidant consumption (vitamin A, C, E, β-carotene, β-cryptoxanthin, lycopene, lutein, and zeaxanthin) and the risk for developing POAG was found. Only recently an investigation of 1,155 women with osteoporotic fractures proposed that a higher intake of certain fruits and vegetables may be associated with a decreased risk of glaucoma [11]. The Ocular Hypertension Treatment Study (OHTS), initiated by the US National Institute of Health, has clearly shown that the lowering of intraocular pressure (IOP) is the one variable factor able to decrease the risk for developing POAG. Topical ocular hypotensive medication was effective in significantly delaying or preventing the onset of POAG in individuals with elevated IOP [12].

Specific flavonoids that are well studied for improving micro-vascular functions may also exert beneficial effects on IOP. The effect of a proprietary flavonoid composition, Mirtogenol^®^ (registered trademark of Indena, Milan, Italy and Horphag Research, London, UK), in subjects with an elevated IOP without glaucoma and with normal vision was studied in a six month trial.

## Methods

Thirty-eight subjects were studied and divided into two groups. The Mirtogenol^®^ group, consisting of 20 subjects aged 44.8±6.5 years (12 males and 8 females), were treated with Mirtogenol^®^. The control group, consisting of 18 subjects aged 44.7±9.2 years (9 males and 9 females), were followed up without treatment. All subjects had complete eye exams, ocular hypertension, and no signs of glaucoma. Their IOP ranged from 22 mmHg to 26 mmHg, and they were not under treatment for the elevated IOP. They all had a cup to disk ratio of less than 0.5, a central corneal thickness greater than 555 microns, and no visual field defects. Subjects with existing POAG or other degenerative eye disorders were excluded. Subjects with cardiovascular diseases requiring medical intervention and those who had any surgery, radiotherapy, or chemotherapy in the last three months were also excluded. Subjects who were pregnant, breast feeding, or planning conception were excluded as well. All the subjects were informed about the aim of the study and treatment procedure according to the Declaration of Helsinki and gave their written informed consent for participation in this investigation. An approval by the ethics committee was not required for this investigation as the subjects were healthy with moderately elevated IOP. The subjects were allowed to take multi-vitamin tablets and any other kind of supplements without restriction.

The patients in the treatment group were given Mirtogenol^®^, one in the morning (AM) and one in the evening (PM), for six months. The Mirtogenol^®^ tablets contained 40 mg of French maritime pine bark extract, Pycnogenol^®^ (Horphag Research, London, UK), and 80 mg of Mirtoselect^®^ standardized bilberry extract (Indena, Milan, Italy). In total, the daily dosage was 80 mg of Pycnogenol^®^ and 160 mg of Mirtoselect^®^. The control group remained untreated. The IOP was measured with the standard Goldmann applanation tonometer at the same time in the morning. Measurements were all performed by the same person to rule out any variations from one investigator to another. At each visit, the IOP was measured in triplicate and mean values were recorded. Visual acuity was obtained using the Snellen chart. Color Doppler imaging (CDI) was used to measure the peak systolic flow velocity (PSFV) and the end diastolic flow velocity (EDFV) of the ophthalmic artery (OA), central retinal artery (CRA), and the posterior ciliary artery (PCA) as previously described [13].

Non-parametric statistics (Mann–Whitney U-test) were used to evaluate the data since the distribution of the IOP and the ocular blood flow were not normal and no standard data was available for these patients. A group of at least 15 subjects in each group was considered a minimal requirement for evaluating variations in the IOP and ocular blood flow, overcoming individual variations, and detecting at least a 5% variation in the evaluated item.

## Results

The Mirtogenol^®^ group (20 subjects) and the control group (18 subjects) were comparable for age (44.8±6.5 years versus 44.7±9.2 years, respectively) and male to female ratio (12:8 versus 9:9, respectively).

**Figure 1:**
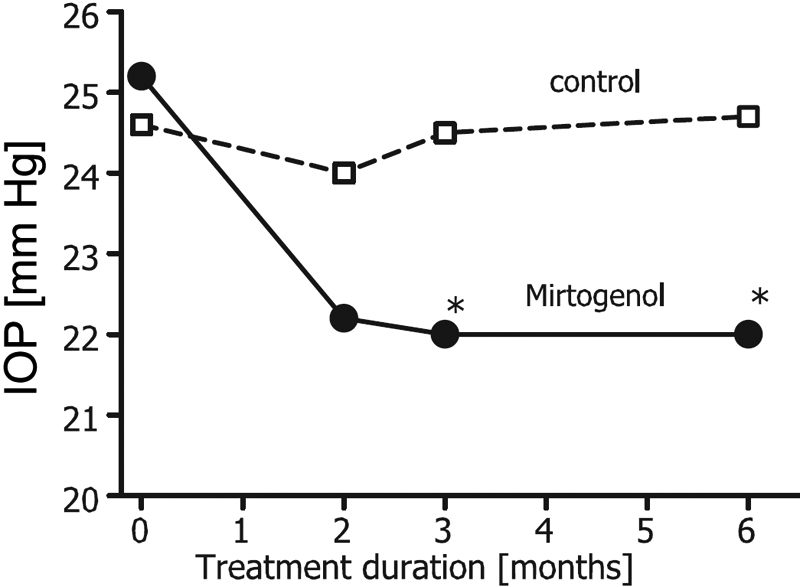
The development of intraocular pressure in subjects treated with Mirtogenol^®^. The asterisk indicates statistical significant difference to the baseline as well as control (p<0.05).

The baseline IOP and standard deviation in the treatment (Mirtogenol^®^) group (25.2±3.1 mmHg) was comparable to the IOP in the controls (24.6±2.8 mmHg). Evaluation measurements were made at two months. A decrease in IOP to 22.2±2.1 mmHg in the Mirtogenol^®^ group was observed. Only a minor decrease to 24.0±2.2 mmHg was found in the controls. The IOP after three months of treatment with Mirtogenol^®^ was 22.0±2.6 mmHg, which was statistically significantly less than at baseline as well as significantly less than the value of 24.5±2.3 mmHg in the controls (p<0.05). Another measurement of IOP was performed after six months, which did not indicate a further lowering of IOP in the Mirtogenol^®^ group (22.0 mmHg ± 2.3) and showed no effect in controls (24.7±2.2 mmHg; Figure 1). Nineteen out of twenty patients in the Mirtogenol^®^ group showed a reduced IOP whereas only 1 of the 18 patients in the control group showed a lowered IOP after the six-month investigation period. During the course of the study, none of the patients in either group had an increased IOP, which would have prompted immediate termination of the patient’s participation in the trial. The visual acuity was normal at trial start and stayed normal in both groups (data not shown).

CDI was obtained from the patient’s ophthalmic, central retinal, and posterior ciliary arteries. The baseline maximum flow velocity (systolic component) was defined at inclusion as 100% for comparison with flow velocities obtained in response to treatment. As presented in Table 1, both the systolic and diastolic components of all three ocular arteries improved (increased), reaching significance after three and six months (p<0.05). This finding suggests an improved ocular arterial blood flow and consequently better tissue oxygenation with Mirtogenol^®^. No significant changes of ocular blood flow occurred during the investigation period in the control group (data not shown). None of the subjects reported side effects, and drop outs did not occur during the trial.

**Table 1:** Color Doppler velocity measurements of ocular arteries in response to Mirtogenol^®^.

		**Baseline**	**2 months**	**3 months**	**6 months**
			**compared to baseline**		
**Central retinal artery**	systolic	100%	+13%	+21%	+20%
	diastolic	23%	+34%	+39%	+38%
**Ophthalmic artery**	systolic	100%	+22%	+22%	+23%
	diastolic	15%	+19%	+25%	+25%
**Posterior ciliary artery**	systolic	100%	+22%	+21%	+22%
	diastolic	14%	+24%	+24%	+23%

The mean systolic component of subjects at the baseline before treatment is set to 100% for comparison with the diastolic component as well as subsequent systolic values found after two, three, and six months of treatment with Mirtogenol^®^. The increased arterial flow velocity was statistically significant after three and six months compared to the baseline as well as the control group values (p<0.05). In the case of controls, no alterations of flow velocity were found (data not shown).

## Discussion

Our objective was to identify a suitable intervention with the potential for protection against developing glaucoma. The major variable risk factor for developing glaucoma is an elevated IOP; therefore, this parameter was singled out as a suitable target for improvement by nutritional means. IOP is regulated by the balance between the secretion and drainage of aqueous humor. The biomechanical parameters and fluid hydrodynamics of the aqueous outflow pathways are complex and not fully understood [14]. This is due to the technical challenges of the unique ocular anatomy with minute amounts of tissue available for study and the difficulties in studying these tissues in vivo. The exact site and nature of a resistance of the outflow system is uncertain.

Many classes of drugs are currently used for lowering IOP in patients with POAG. Mostly applied as eye drops they may reduce the production of aqueous such as in the case of beta blockers, alpha-adrenergic agents, and carbonic anhydrase inhibitors. The widely used prostaglandin analogs (e.g., “latanoprost”) increase uveoscleral outflow probably by rendering capillaries more absorptive as a result of stimulated matrix metalloproteinase secretion. Inflammatory manifestations in human conjunctiva were described for the long-term use of prostaglandin analogs [15]. Interestingly, the exact method of action of the commonly applied beta-blocker, timolol, remains unknown. Timolol appears to not significantly alter the blood aqueous barrier as shown with fluorescence angiography [16]. Essentially, all these eye drops for intraocular hypertension are known to cause considerable local and systemic side effects [17].

We speculate that a vascular function could play a significant role in the pathomechanisms underlying high IOP. There are observations suggesting an involvement of an impaired vascular response for an increased IOP [18]. The well known increase of IOP in the supine position is more pronounced and develops faster in aging healthy people than in young healthy people, suggesting less resistance to hydrostatic pressure with older age [19]. This is expected to result from an increased aqueous release from the ciliary body. Pharmacologic studies have previously described that Mirtoselect^®^ counteracts the hyperpermeability of ciliary capillaries, initiated by paracentesis, as measured by the Evans Blue concentration in the aqueous humor [20]. Furthermore, as recently reviewed, anthocyanosides contained in the standardized bilberry extract are the only flavonoids able to reach the eye as a target organ in experimental animals. Unchanged anthocyanosides demonstrated after oral administration that it is absorbed and distributed into ocular tissues, showing its ability to pass through the blood-aqueous and blood retinal barriers [21].

Mirtoselect^®^ as a consequence has been suggested to be useful as a therapeutic application for ophthalmic problems involving the altered blood-aqueous barriers [22].

In retinopathy patients, treatment with Mirtoselect^®^ had a significant reduction of retinal micro-hemorrhages, and a regression of localized retinal edema was found using fundus fluorescence angiography [23]. The effect of bilberry extract on the ocular micro-circulation was investigated in exploratory studies using electroretinography in patients with myopia and glaucoma [24]. Pycnogenol^®^ likewise was shown to improve pathologic permeability of blood vessels [25] and has been extensively studied for enhancing capillary resistance and integrity in retinopathy [9].

There is increasing evidence of the contractile activities of the trabecular meshwork, which allows for regulatory mechanisms controlling fluid outflow. Trabecular meshwork cells exhibit smooth muscle-like contractile properties, which respond to the vasoconstrictory peptide, endothelin-1 [26]. Endothelin-1 has been identified in elevated quantities in the aqueous humor of patients with POAG. Furthermore, there is experimental evidence for endothelial nitric oxide being involved in the regulation of ocular vascular tone. In healthy volunteers, infusion of nitric synthase inhibitor N-monomethyl-L-arginine (L-NMMA) significantly reduced pulsatile choroidal and total choroidal blood flow [27]. Pycnogenol^®^ has previously been shown to significantly enhance the generation of endothelial nitric oxide in healthy humans [28]. Pycnogenol^®^ has also been found to significantly lower plasma endothelin-1 in patients with type II diabetes [29]. Mirtoselect^®^ bilberry extract was found to stimulate arteriolar vasomotion in preclinical tests [30]. In our study, we were indeed able to demonstrate an improved blood flow velocity of patient’s ophthalmic artery, central retinal artery, and posterior ciliary arteries with Mirtogenol^®^.

It is not known whether Mirtogenol^®^ affects outflow pathways or aqueous humor inflow or both. This will remain very difficult to identify. Methods such as the “water-drinking test,” which involves a significant IOP increase subsequent to drinking large quantities of water, might offer some insight. It was suggested that medications, which predominantly enhance outflow such as prostaglandin analogs, provide better IOP stabilization in such experiments [31].

Our study is the first demonstration showing that dietary intervention can help to control IOP and increase ocular blood flow in asymptomatic subjects and if taken in time, may prevent an evolution to higher pressure and symptomatic glaucoma. The safety of Mirtogenol is warranted as its components Mirtoselect and Pycnogenol are used as dietary supplements since decades without serious side effects. A clinical trial with a larger number of subjects should further assess the benefits of Mirtogenol for controlling IOP.
